# Pyroptosis and adaptive immunity mechanisms are promptly engendered in mesenteric lymph-nodes during pig infections with *Salmonella enterica* serovar Typhimurium

**DOI:** 10.1186/1297-9716-44-120

**Published:** 2013-12-05

**Authors:** Rodrigo Prado Martins, Carmen Aguilar, James E Graham, Ana Carvajal, Rocío Bautista, M Gonzalo Claros, Juan J Garrido

**Affiliations:** 1 Grupo de Genómica y Mejora Animal, Departamento de Genética, Facultad de Veterinaria, Universidad de Córdoba, Campus de Rabanales, Edificio Gregor Mendel C5, 14071, Córdoba, Spain; 2 Department of Microbiology and Immunology, University of Louisville, School of Medicine, 40202, Louisville, KY, USA; 3 Departamento de Sanidad Animal, Facultad de Veterinaria, Universidad de León, 24071, León, Spain; 4 Plataforma Andaluza de Bioinformática, Universidad de Málaga, Parque Tecnológico de Andalucía, 29590, Málaga, Spain

## Abstract

In this study, we explored the transcriptional response and the morphological changes occurring in porcine mesenteric lymph-nodes (MLN) along a time course of 1, 2 and 6 days post infection (dpi) with *Salmonella* Typhimurium. Additionally, we analysed the expression of some *Salmonella* effectors in tissue to complete our view of the processes triggered in these organs upon infection. The results indicate that besides dampening apoptosis, swine take advantage of the flagellin and prgJ expression by *Salmonella* Typhimuriun to induce pyroptosis in MLN, preventing bacterial dissemination. Furthermore, cross-presentation of *Salmonella* antigens was inferred as a mechanism that results in a rapid clearance of pathogen by cytotoxic T cells. In summary, although the *Salmonella* Typhimurium strain employed in this study was able to express some of its major virulence effectors in porcine MLN, a combination of early innate and adaptive immunity mechanisms might overcome virulence strategies employed by the pathogen, enabling the host to protect itself against bacterial spread beyond gut-associated lymph-nodes. Interestingly, we deduced that clathrin-mediated endocytosis could contribute to mechanisms of pathogen virulence and/or host defence in MLN of *Salmonella* infected swine. Taken together, our results are useful for a better understanding of the critical protective mechanisms against *Salmonella* that occur in porcine MLN to prevent the spread of infection beyond the intestine.

## Introduction

Infections by *Salmonella* are a major health problem in the developing and developed world. In the European Union, despite the current decreasing trend of human cases, *Salmonella* persists as the main cause of food-borne outbreaks [1]. Pork is considered to be a significant source of *Salmonella* to humans next to eggs and poultry meat [2]. Indeed, according to the European food safety authority (EFSA), *Salmonella enterica* serovar Typhimurium (herein *Salmonella* Typhimurium) is the second serovar most frequently reported in human salmonellosis and infection by this pathogen is mostly associated with the consumption of contaminated pork [1].

Since the food industry and direct contact with infected animals represent the main sources of non-typhoid *Salmonella*[3], prevention of human salmonellosis depends significantly on decreasing the prevalence of infection in livestock hosts [4]. *Salmonella* Typhimurium infected pigs generally carry this serotype asymptomatically in the tonsils, intestines and gut-associated lymphoid tissue, posing an important threat to animal and human health [5]. Epidemiological studies assert that *Salmonella* prevalence in slaughter swine lymph nodes varies widely at the country level, ranging from0 to 29% [2]. Although salmonellosis in pigs has been the subject of intensive research [5], a thorough knowledge of the pathogenesis of porcine infections with broadhost range *Salmonella* serotypes is still necessary. A combination of system-wide approaches and in vivo infection models is expected to generate precise and novel data to analyze the response to *Salmonella* infections in pigs [6]. Thus, whole-genome expression analysis has been used to explore gene expression changes during infection of pigs by *Salmonella*, contributing to identify molecules and pathways associated with the host response to infection [7,8]. More recently, proteomic techniques have also been employed as a step towards a detailed understanding of the disease mechanisms [9,10]. However, despite this, there is a need to deepen understanding of the biological processes that control host-pathogen interaction and *Salmonella* persistence in porcine lymphatic tissue, which could provide new targets for treatment and control of salmonellosis in this species. Therefore, the objective of the current study was to explore the early transcriptional response of porcine mesenteric lymph-nodes (MLN) to *Salmonella* Typhimurim using a time-course analysis of an in vivo infection. In addition, the expression of some pathogen virulence effectors, as well as the morphological alterations associated with the presence of the bacteria in the tissue were also evaluated.

## Materials and methods

### Experimental infection and tissue sampling

Sixteen crossbred weaned piglets of approximately four weeks of age, serologically and fecal-negative for *Salmonella* were used in an experimental infection described elsewhere [11]. Briefly, twelve piglets were orally infected with 10^8^ cfu of a *Salmonella* Typhimurium phagetype DT104 strain isolated from a naturally infected pig [11], whereas the control group (4 animals) received sterile medium. Non-infected control pigs were necropsied prior to the experimental infection (0 day post-infection – dpi) and four randomly chosen infected piglets were necropsied at 1, 2 or 6 dpi. Samples of MLN were collected from all experimental animals and immediately frozen in liquid nitrogen for RNA and protein isolation or fixed in 10% neutral buffered formalin for histological processing. All procedures involving animals were performed in accordance with the European regulations regarding the protection of animals used for experimental and other scientific purposes. Piglets were housed in experimental isolation facilities of the University of Leon (Spain). Animal care and procedures were in accordance with the guidelines of the Good Experimental Practices (GEP), under the supervision of the Ethical and Animal Welfare Committee of the University of Leon (Spain).

### RNA purification

After treatment with RNAlater-ICE (Ambion, Inc, Austin, TX, USA), MLN samples were soaked in RLT buffer (Qiagen, Valencia, CA, USA) and disrupted in a rotor-stator homogenizer. RNA was isolated using the AllPrep DNA/RNA/Protein Mini Kit (Qiagen), digested with the RNase-Free DNase Set (Qiagen) according to the manufacturer’s instructions and routinely precipitated with ethanol. RNA integrity was evaluated using the Experion RNA automated electrophoresis system (Bio-Rad, Hercules, CA, USA) before being quantified using a ND-1000 spectrophotometer (Nanodrop Technologies, Wilminton, USA).

### Microarray analysis

Gene expression analysis was carried out using the GeneChip Porcine Genome Array of the Affymetrix platform (Affymetrix Inc., Santa Clara, CA, USA) at the Genomics Unit of CABIMER (Andalusian Center for Molecular Biology and Regenerative Medicine, Seville, Spain). This chip contains 23 937 probe sets to interrogate 23 256 transcripts in the pig, which represents 20 201 genes. The One-Cycle Eukaryotic Target Labeling Assay was used to obtain biotinylated cRNA to be used in the subsequent chip hybridization according to the manufacturer’s instructions (Expression Analysis Technical Manual, Affymetrix). The biotinylated cRNA targets were then cleaned up, fragmented, and hybridized with the GeneChip Porcine Genome Array following Affymetrix recommended protocols. Chips were washed, stained with a GeneChip Fluidics Station 450 (Affymetrix) using the standard fluidics protocol and scanned with an Affymetrix GeneChip Scanner 3000 (Affymetrix). Probe signal intensities were captured and processed with the GeneChip Operating Software 1.4.0.036 (Affymetrix) and the resulting CEL files were reprocessed using robust multi-array average normalization (RMA) [12]. Because the aim of analysis was to detect changes in gene expression along a time-course of infection, differentially expressed (DE) genes were accessed by the BATS (Bayesian Analysis of Time Series) software package [13], using default settings. A Bayes Factor (BF) value of 0.05 was used as cutoff to rank significantly regulated transcripts. Since the Affymetrix Porcine GeneChip is not fully annotated in all the features, it was re-annotated with Blast2GO [14] with a minimum E-value of 10^-10^ and a minimum similarity of 50%.

### Systems biology analysis

The list of genes that showed significant changes in expression was uploaded into Ingenuity Pathway Analysis (IPA, Ingenuity Systems Inc, Redwood City, CA, USA) [15] for bioinformatics analysis. Additionally, the DAVID Bioinformatic Database [16] was used applying the default settings to refine some data from IPA analysis. Gene interaction networks were automatically generated, ranked by score and depicted on IPA as follows: each node in the network diagram represented a gene and its relationship with other molecules was represented by a line (solid and dotted lines represent direct and indirect association respectively). Nodes with a red background were input genes detected in this study while grey nodes were molecules inserted by IPA based upon the Ingenuity Knowledge Base to produce a highly connected network. The score estimated the probability that a collection of genes equal to or greater than the number in a network could be achieved by chance alone. Scores of 3 or higher were considered to have a 99.9% confidence of not being generated by random chance alone. For statistical analysis of enriched functions/pathways, an IPA Knowledge Base was used as a reference set and the Fisher’s exact test was employed to estimate the significance of association. *P*-values below 0.05 were considered statistically significant. For graphical representation of the canonical pathways, the ratio indicates the percentage of genes taking part in a pathway that could be found in an uploaded data set and –log(*p*-value) means the level of confidence of association. The threshold line represented a *p*-value of 0.05.

### Relative gene expression analysis by qPCR

Real-time quantitative PCR (qPCR) assays were performed as previously described [11]. Fold change values were calculated by the 2^−ΔΔCq^ method [17] using beta-actin as the reference gene. Afterwards, data were standardized as proposed by Willems et al. [18] and analyzed by Kruskal–Wallis and Mann–Whitney tests using the software SPSS 15.0 for Windows (SPSS Inc, Chicago, IL, USA). Fold changes of 1 denoted no change in gene expression. Values lower and higher than 1 denoted down and up-regulation respectively. To be represented in Table 1, a fold change of down-regulated genes was calculated as −1/2^−ΔΔCq^. Primer pairs used for amplifications can be found as supporting information (see Additional file 1).

**Table 1:** Microarray data validation by qPCR.

**Gene**	**MICROARRAY**				**qPCR**			
	**Fold change**			**BF**	**Fold change**			***p*-value**
	**1 dpi**	**2 dpi**	**6 dpi**		**1 dpi**	**2 dpi**	**6 dpi**	
CD180	1.7	2.6	1.5	0.0000429	1.1	1.8	1.2	0.010
CD1A	1.1	−1.4	1.2	0.00047793	−1.4	−2.5	1.2	0.013
DAB2	−1.2	−2.6	−1.2	6.62E-13	−3.1	−6.5	−2.6	0.001
EIF4H	−1.1	−1.1	−1.1	0.0000101	−1.5	−1.4	−1.8	0.021
ENPP6	1.3	2.0	−1.2	0.0000448	1.2	1.8	−1.7	0.000
F13A1	1.4	2.2	−1.1	0.00000227	1	1.7	−2.2	0.012
HLA-B^b^	1.0	−1.1	−1.2	0.00023747	−1.4	−1.4	−1.9	0.047
HLA-DRB5^b^	1.0	−1.1	1.0	0.0000311	−1.4	−1.6	−2	0.036
HSPA1B^a^	3.3	1.4	−1.1	0.0001166	2.5	1.4	−1.3	0.025
HSPH1	2.3	1.7	−1.0	0.00000424	1.5	1.1	−2	0.003
IL16	−1.0	−1.2	−1.1	8.12E-07	1	−1.1	−1.5	0.035
LPCAT2	1.2	2.3	1.0	0.0000146	1.4	2	−1.3	0.010
PSMC2	−1.0	−1.0	−1.1	0.00105861	−1.1	−1.4	−1.8	0.036
TRAC	−1.0	−1.1	−1.1	0.00000951	−1.5	−1.8	−1.8	0.010

^a^Data from microarray analysis are mean values from two different probes. ^b^Amplified with SLA-B and SLA-DRB5 primers.

### Western blot analysis

For protein extractions, MLN samples from all experimental animals were separately homogenized on ice with lysis buffer (7 M urea, 2 M thiourea, 4% w/v CHAPS, 0.5 mM PMSF) using a glass tissue-lyser and protein lysate concentration was determined using a Bradford Protein Assay (Bio-Rad). Subsequently, protein from individual replicates belonging to the same group was pooled (30 ug total), electrophoretically fractionated in 12% (w/v) SDS-PAGE gels and transferred onto a PVDF membrane (Millipore, Bedford, MA, USA). Western blot assays were carried out as described by Martins et al. [10] employing the following primary antibodies: 4B7/8 for swine histocompatibility class I antigen (SLAI) detection [19], 1 F12 for swine histocompatibility class II antigen (SLAII) detection [19], anti-CTLA4 (Epitomics, Burlingame, CA, USA) and anti-Clathrin light chain (ab24579, Abcam, Cambridge, UK). To confirm equal sample loading, membranes were reblotted with anti-GAPDH monoclonal antibody (GenScript, Picastaway, NJ, USA) and no statistical differences for GAPDH abundance were observed between groups in all assays. Membranes were scanned in an FLA-5100 imager (Fujifilm, Tokyo, Japan) and signal intensity was determined using Multigauge software (Fujifilm, Tokyo, Japan) as previously described [10].

### Histopathology, immunohistochemistry and confocal microscopy analysis

Paraffin sections (5 μm) of formalin fixed samples were routinely processed and stained with hematoxylin and eosin (H&E) to evaluate tissue morphology. For immunohistochemistry assays, a standard avidin-biotin peroxidase method was performed as described elsewhere [20] employing 1 F12 monoclonal antibody and a biotinylated anti-mouse Ig (Dako, Barcelona, Spain) as a secondary antibody. Immunofluorescence using confocal microscopy was performed employing the anti-SLAII 1 F12 monoclonal antibody, a rabbit polyclonal antibody against the *Salmonella* somatic (O4, 5, 12) antigen [10] and a rabbit polyclonal antibody anti-*Salmonella* Typhimurium flagellin [21]. Fluorescein isothiocyanate (FITC)-conjugated goat anti-rabbit IgG (Sigma-Aldrich, St. Louis, MO, USA) and Alexa Fluor 594 anti-mouse IgG (Life Technologies, Carlsbad, CA, USA) were used as secondary antibodies. Immunostaining was performed as described by Robertson et al. [22]. Briefly, deparaffinized sections of formalin fixed MLN were blocked for 30 min with 1% bovine serum albumin and 2% foetal calf serum in PBS. Then, sections were incubated overnight at 4 °C with primary antibodies, washed three times with PBS for 5 min and incubated for 1 h at 37 °C with fluorescent secondary antibodies. For negative controls, primary antibody was omitted. Finally, sections were washed three times for 5 min in PBS containing 1.43 μM 4′,6-diamidino-2-phenylindole (DAPI, Life Technologies). Samples were subsequently evaluated and imaged using an LSM 5 Exciter confocal microscope (Carl Zeiss, Jena, Germany).

### Cell death analysis

Formalin fixed MLN samples were evaluated for cell death by terminal deoxynucleotidyl transferase dUTP nick end labeling (TUNEL), employing the TUNEL Apoptosis Detection Kit for Paraffin-embedded Tissue Sections (GenScript, Picastaway, NJ, USA) according to the manufacturer’s instructions. Briefly, proteinase K treated samples were permeabilized with 0.1% Triton X-100 and 0.1% sodium citrate for 10 min and incubated with Blocking Solution II (GenScript) for 30 min. Subsequently, tissues were covered with 50 μL of TUNEL Reaction Mixture (GenScript), incubated at 37 °C for 1 h in a dark humidified chamber and washed in PBS. Sections were examined in an LSM 5 Exciter confocal microscope (Carl Zeiss MicroImaging GmbH, Jena, Germany) using excitation wave 450–500 nm and emission wave 515–565 nm (green). Fluorescence intensity was quantified with the ImageJ software 1.46r [23] and data were analyzed by ANOVA (*p*-value cutoff of 0.05) using SPSS 15.0 for Windows (SPSS Inc).

### Selective capture of transcribed sequences (SCOTS)

Selective capture of *Salmonella* transcripts from MLN of pigs at 2 dpi was performed by the SCOTS method [24], following the procedure described by Sheikh et al. [25]. Briefly, 5 μg of total RNA from infected MLN samples was converted into first strand cDNA by using random priming and Superscript III reverse transcription (Life Technologies). Subsequently second strand cDNA was produced employing DNA polymerase I (Klenow fragment, Life Technologies). To create a corresponding in vitro *Salmonella* Typhimurium cDNA sample for comparison, the same bacterial isolate employed in the experimental infection was grown to early-log growth phase (OD_600_ = 0.3) and late-log growth phase (OD_600_ = 0.8) in Luria Bertani (LB) broth. Afterwards, *Salmonella* Typhimurium transcripts were selectively captured from in vivo and in vitro double stranded cDNA by hybridization to sonicated biotinylated genomic *Salmonella* DNA, which was previously blocked with *Salmonella* ribosomal DNA fragments. Microbial cDNA-genomic DNA hybrids were then captured by binding to streptavidin-coated beads (Dynabeads M-280 streptavidin, Invitrogen) and bacterial transcripts were eluted by alkaline denaturation. Eluted bacterial cDNA was then PCR-amplified with conserved primers and finally purified using Qiagen PCR column purification kit (Qiagen). After that, one round captured and purified cDNA from both in vitro and in vivo conditions were quantified by spectrophotometry and used as template (10 ng) for qPCR assays as described above. Primer pairs used for amplifications can be found as supporting information (see Additional file 2). Gene expression levels were estimated employing *gyrA* as the reference gene. Since tissue from uninfected pigs was negative for *Salmonella,* those samples could not be used as reference for fold change calculations of pathogen gene expresion. In addition, most screened genes showed Cq values inferior to those observed for *gyrA* in infected MLN. For these reasons, gene expression levels were alternatively estimated as follows: *gyrA* Cq – target gene Cq. Higher values meant higher expression levels and vice-versa.

## Results

### Transcriptional changes in porcine MLN upon *salmonella* Typhimurium infection and data validation

Microarray technology coupled to a Bayesian analysis was employed to explore the transcriptional response of porcine MLN to *Salmonella* Typhimurium along a time course of 1, 2 and 6 dpi. BATS, a method specifically designed for the analysis of time series microarray data [10], revealed significant changes in expression (BF < 0.05) for 290 transcripts, representing 285 unique genes, as a result of the bacterial challenge (see Additional file 3). Then, to validate data, qPCR assays were performed on a panel of fourteen genes identified by BATS analysis. As expected, all of them were confirmed to be significantly regulated (*p* < 0.05) after infection (Table 1). Furthermore, an identical expression trend was observed for most screened genes by qPCR and microarray analysis.

### Biological interpretation of microarray data

**Figure 1:**
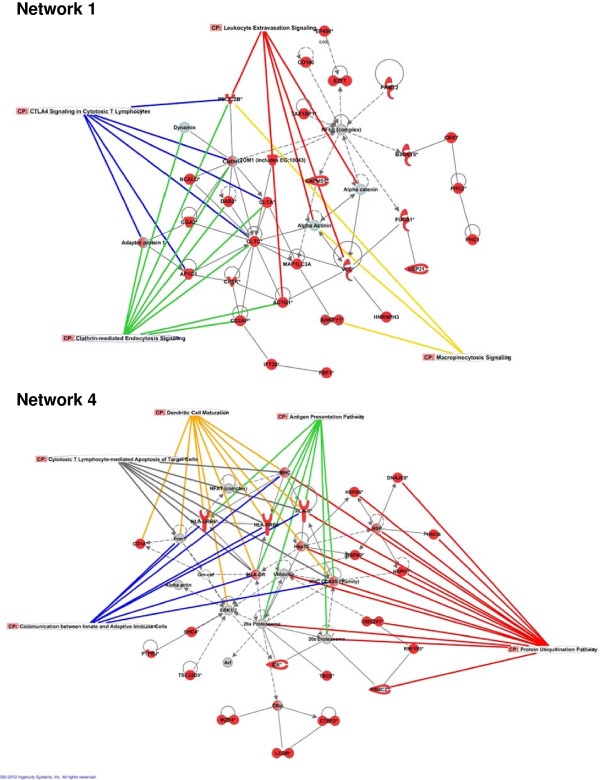
**Gene network analysis by Ingenuity Pathway Analysis (IPA).** Visual representation of networks 1 and 4. Red and grey nodes are input and IPA-inserted molecules respectively. Colored lines highlight genes that take part in an enriched Canonical Pathway.

**Figure 2:**
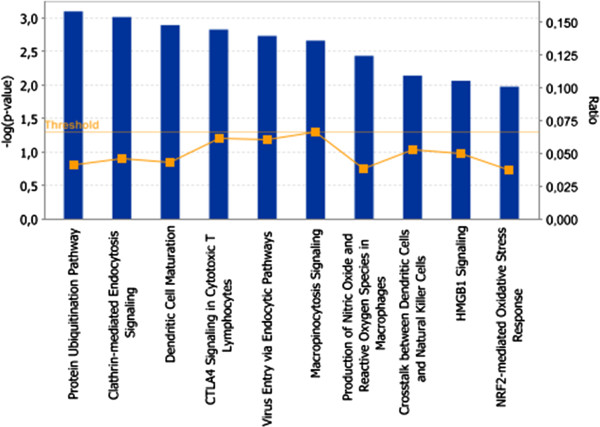
**Top 10 enriched canonical pathways.** Blue bars and yellow squares denote –log(*p*-value) and ratio respectively. Threshold line represents a *p*-value of 0.05.

To translate microarray data into functional biological information, bioinformatics tools were employed to gain an insight into networks, functions and pathways associated with the transcriptomic response of porcine MLN to *Salmonella* Typhimurium (see Additional file 4)*.* IPA analysis generated 17 gene interaction networks integrated by molecules associated with mechanisms such as cell-mediated immune response, cell-to-cell signaling and interaction, tissue morphology, cell movement and cell death. Networks 1 and 4 (Figure 1) revealed direct relationships between molecules taking part in five of the ten top enriched canonical pathways after infection (Figure 2). Moreover, network 4 demonstrated a central role for heat shock proteins and MHC encoding genes in the establishment of different mechanisms carried out in MLN in response to *Salmonella* Typhimurium*.* IPA also ascertained the enrichment of biological functions other than those identified by network analysis (Table 2). Thus, “Inflammatory disease” was the Ingenuity biofunction more significantly related to the differentially expressed genes, followed by “Protein synthesis” and “Antigen presentation”.

**Table 2:** **Top five biological functions enriched in MLN of pigs infected with *Salmonella *** **Typhimurium** ***.***

**Annotations**	***p*-Value**^**a**^	**Input genes (n)**
Inflammatory disease	4.64E-05 – 2.65E-02	13
Protein synthesis	6.52E-05 – 1.86E-02	32
Antigen presentation	1.8E-04 – 1.68E-02	5
Cell death	1.8E-04 – 2.67E-02	78
Cell-to-cell signaling and interaction	1.8E-04 – 2.67E-02	27

^a^*p*-values were calculated with the right-tailed Fisher’s Test.

### Modulation of immune response mechanisms

**Figure 3:**
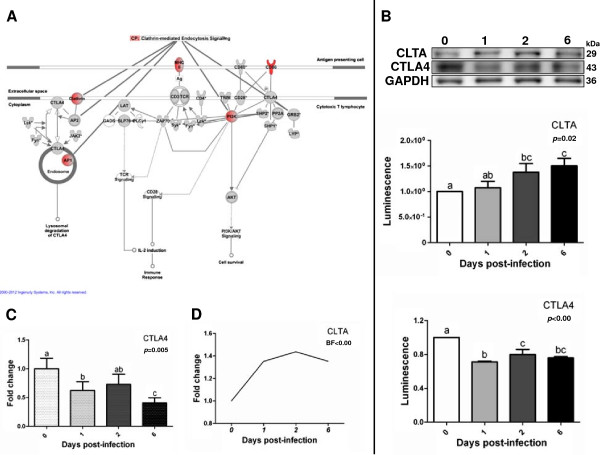
**Interaction between CTLA-4 signaling in cytotoxic T lymphocytes and Clathrin-mediated endocytosis signaling pathways. A**: visual representation of CTLA-4 signaling in cytotoxic T lymphocyte pathway stressing the regulation of molecules taking part in Clathrin-mediated endocytosis signaling. Red and grey nodes are input and IPA-inserted molecules respectively. **B**: Western blot analysis of CTLA4 and CLTA in MLN at 1, 2 and 6 dpi. **C**: mRNA quantification of *CTLA4* by qPCR. **D**: expression pattern of *CLTA* mRNA by microarray analysis.

Wide transcriptomic data analysis by bioinformatics tools revealed an enrichment of distinct mechanisms leading to immune response activation in porcine MLN upon *Salmonella* Typhimurium infection and depicted connections between them. As illustrated in Figure 3A, the association between “CTLA-4 signaling in cytotoxic T lymphocytes” and “Clathrin-mediated endocytosis signaling” pathways was established by the regulation of shared genes. Then, we checked the abundance level of cytotoxic T-lymphocyte-associated protein 4 (CTLA-4) and clathrin light chain A (CLTA) by Western blot, noting that CLTA was more abundant in infected animals whereas CTLA4 showed reduced levels after infection (Figure 3B). Since changes in *CTLA4* expression could not be detected by microarray analysis, we verified *CTLA4* mRNA levels by qPCR (Figure 3C). In accordance with Western blot assays, *CTLA4* was observed to be significantly down-regulated in infected animals at 1 and 6 dpi. Concerning *CLTA*, a similar trend towards up-regulation was observed at mRNA (Figure 3D) and protein levels.

**Figure 4:**
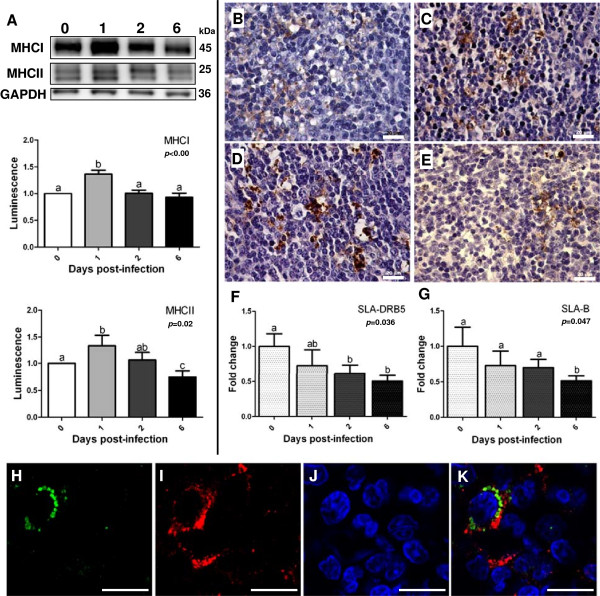
**Analysis of MHC molecules in porcine MLN after *Salmonella *Typhimurium infection. A**: Western blot analysis of MHC class I and II at 1, 2 and 6 dpi. **B-E**: MHCII labeling in tissue at 0 **(B)**, 1**(C)**, 2 **(D)** and 6 **(E)** dpi by immunohistochemistry. Scale bar = 20 μm. **F-G**: mRNA quantification of SLA-DRB5 (MHCII) **(F)** and SLA-B (MHCI) **(G)** by qPCR. **H-K**: confocal analysis of *Salmonella* Typhimurium infected MLN demonstrate the presence of the pathogen in MHCII positive cells. *Salmonella* Typhimurium*-*FITC **(H)**, MHCII-Alexa Fluor® 594 **(I)**, DAPI **(J)** and merge **(K)**.

System biology analysis also revealed the involvement of MHC encoding genes in many processes triggered in MLN in response to *Salmonella* Typhimurium. Thus, changes undergone by these molecules were evaluated employing different approaches. Firstly, Western blot analysis demonstrated that major histocompatibility antigens class I (MHCI) and class II (MHCII) were more abundant in tissue at 1 dpi (Figure 4A). Similarly, immunohistochemistry revealed higher MHCII expression at initial stages of infection, with increased levels of this molecule mainly detected in large irregularly-shaped mononuclear cells (Figure 4B-E). Then, confocal microscopy analysis uncovered the presence of *Salmonella* Typhimurium antigens in cells showing high levels of MHCII (Figure 4H-K), suggesting a connection between the increase of this receptor at the protein level and the presence of pathogen in tissue. Curiously, *MHCI* and *MHCII* (annotated in data sets as HLA-B and HLA-DRB, respectively) were found to be down-regulated by microarray analysis. Since these results were confirmed by qPCR (Figure 4F-G), a divergence between transcriptomic and proteomic changes could be highlighted for these molecules in infected MLN.

### Tissue morphology and cell death

**Figure 5:**
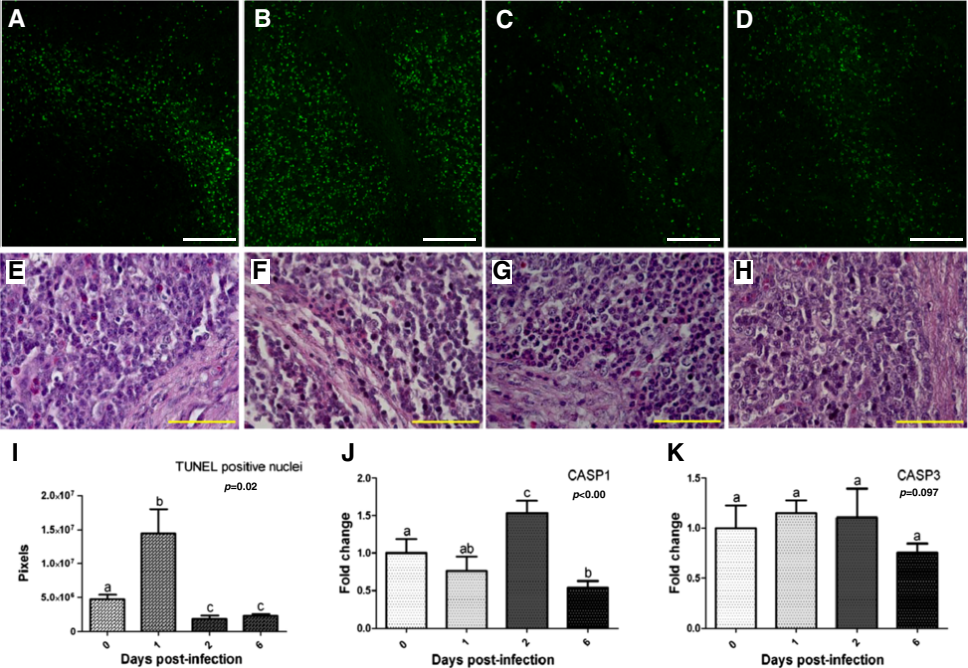
**Cell death and histopathologic analysis of MLN from *Salmonella *Typhimurium infected pigs. A-D**: Terminal deoxynucleotidyl transferase dUTP nick end labeling (TUNEL) analysis at 0 **(A)**, 1**(B)**, 2 **(C)** and 6 **(D)** dpi. Scale bar = 100 μm. **E-H**: H&E staining of at 0 **(E)**, 1**(F)**, 2 **(G)** and 6 **(H)** dpi. Scale bar = 50 μm. **I**: Quantification of TUNEL fluorescent labeling shows an increase of positive nuclei at 1 dpi and a decrease to levels inferior than the controls at 2 and 6 dpi. **J-K**: mRNA quantification of *CASP1***(J)** and *CASP3***(K)** by qPCR.

“Cell death” was one of the most significantly altered biological functions after infection and integrated the highest number of differentially expressed genes (*n* = 78). In order to find GO subcategories associated to genes implicated in this function, a data set arranged into “Cell death” by IPA was loaded into the DAVID Bioinformatic Database. As expected, enriched terms were related to cell proliferation, differentiation and death (see Additional file 5). Among them, processes such as “Negative regulation of apoptosis” and “Antiapoptosis” were found to be enriched all along the infection, suggesting an inhibition of apoptosis. To deepen and sharpen these results, TUNEL analysis followed by confocal microscopy was performed to elucidate the cell death mechanisms induced in MLN after *Salmonella* Typhimurium infection. As shown in Figure 5A-D and 5I, DNA damage detected by TUNEL staining peaked at 1 dpi and decreased at 2 and 6 dpi compared to controls. Afterwards, expression levels of *CASP1* and *CASP3*, the main pyroptosis and apoptosis inducers respectively, were quantified by qPCR. *CASP1* mRNA was significantly up-regulated at 2 dpi and down-regulated at 6 dpi (Figure 5J), whereas no significant changes were observed for *CASP3* (Figure 5K). Finally, lymph-node sections were H&E-stained to analyse the structural changes undergone by tissue as a consequence of infection (Figure 5E-H). Besides the loss of the typical lymph-node micro-architecture, phagocyte infiltration was the main alteration detected after infection, being observed mainly at 1 and 2 dpi.

### *Salmonella* Typhimurium localization and gene expression in vivo

**Figure 6:**
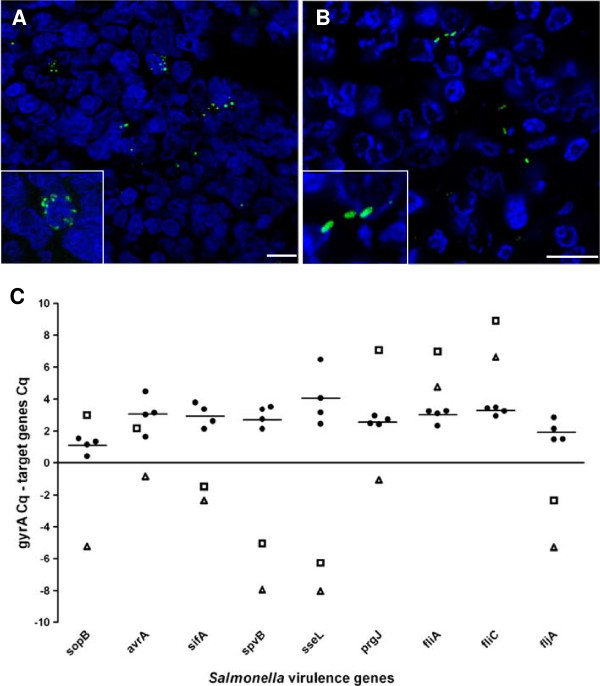
***Salmonella *Typhimurium labeling and gene expression in porcine MLN. A-B**: Different labeling profiles found for *Salmonella* Typhimurium in porcine MLN. Scale bar = 10 μm. **(A)** Pathogen detection as spherical structures in the perinuclear zone of mononuclear cells. **(B)** Staining of bacilli shaped structures. **C**: Analysis of *Salmonella* Typhimurium gene expression by SCOTS in vivo and in vitro. Black dots and bars respectively represent individual and mean expression values from analysis of cDNA from pig infected MLN. Triangles (early logarithmic phase) and squares (late logarithmic phase) denote gene expression data from *Salmonella* Typhimurium cultures. Higher values mean higher expression levels and vice-versa.

*Salmonella* Typhimurium was detected in MLN along the whole studied time course, with higher *Salmonella* levels being observed at 2 dpi (data not shown). Confocal microscopy analysis revealed two distinct bacterial populations according to location and labeling. As shown in Figure 4H-K and Figure 6A, some bacteria were labeled as spherical structures located in the perinuclear zone of mononuclear cells. In addition, regular bacilli shaped structures showing a different distribution were also detected (Figure 6B). Although Z-stack confocal images indicate that, most likely, this second population was found in an extracellular environment (see Additional file 6), further experiments will be necessary to clarify whether the pathogen is in the cytosol or outside the cell. Ultimately, expression of some *Salmonella* genes in vivo was also studied by the SCOTS approach and compared to in vitro conditions. Type III secretion systems (TTSS) encoded genes (*sopB, avrA, sifA, sseL and prgJ*) and *spvB* were found to be expressed by *Salmonella* Typhimurium in porcine MLN. Notably, lower expression of these genes was detected in vitro when compared to in vivo. The results regarding genes coding for flagella components and regulators show higher expression levels in vitro for *fliA, fliC* whereas *fljA* mRNA was observed to be more expressed in vivo (Figure 6C).

## Discussion

Gut-associated lymphoid tissues have been proved to be an important niche for *Salmonella* during pig infections. Previous reports stated that *Salmonella* Typhimurium can be found in MLN of infected pigs from 2 h [5] up to 6 weeks after oral inoculation [26] and sustain these organs as immune inductive sites during pig salmonellosis [6,7,10,26,27]. For this reason, in this work we aimed at dissecting host response mechanisms occurring in porcine MLN upon interaction with *Salmonella* Typhimurium. Additionally, expression of some *Salmonella* virulence effectors was also analyzed in infected tissues attempting to integrate information from both the host and pathogen.

The systems biology analysis reported in this study demonstrates the involvement of MHC molecules in several mechanisms triggered in swine MLN after bacterial challenge. Intriguingly, both MHCI and MHCII encoding genes were found to be down-regulated all along the studied time course, in spite of the increased levels observed for these receptors at 1 dpi by Western blot and microscopic analysis. We envisage that initially, antigen presenting cells bearing high levels of MHC migrate to MLN leading to an increase of these receptors in tissue, as suggested by the detection of *Salmonella* antigens in cells showing high levels of MHCII. Subsequently, processes carried out in MLN might reduce MHC mRNA and protein expression levels at later times of infection. Previous studies demonstrate that *Salmonella* interferes with antigen presentation by reducing MHCII surface expression via a mechanism dependent on the *Salmonella* pathogenicity island (SPI)**-**2 encoded effector SifA [28-30]. It is noteworthy that in this study we show that *sifA* is expressed by *Salmonella* Typhimurium in porcine MLN. Besides, it has been previously observed by us [21] and others [27] that pig infections with *Salmonella* Typhimurium do not produce an up-regulation of cytokines involved in T helper 1 (Th1) response in MLN, on the contrary to previous reports in mice [31]. These findings could be related to the ability of pathogens to limit antigen presentation to CD4 restricted T cells by reducing MHCII levels in infected cells. *Salmonella* removes mature MHCII complexes from the cell surface by enhancing their ubiquitination in a clathrin and AP2-dependent way [29]. Curiously, we identified the “Protein ubiquitination pathway” and “Clathrin-mediated endocytosis signaling” as the most significantly affected canonical pathways upon infection. In spite of the reported use of clathrin-mediated endocytosis in bacterial-induced internalization, *Salmonella* is not able to employ this machinery to invade [32]. So, instead of promoting direct entry of the pathogen to host cells, enrichment of clathrin-mediated endocytosis could be related to the modulation of MHCII expression by *Salmonella* found in tissue. Therefore, this evidence as a whole could suggest a hampering of processes mediated by MHCII in swine MLN following *Salmonella* infection.

On the contrary, network analysis also associated “Clathrin-mediated endocytosis signaling” to “CTLA4 signaling in cytotoxic T lymphocytes’ pathway”, bringing to light a possible role of the former process in adaptive immunity triggering. CTLA4, an important negative regulator of the T cell immune response [33], is endocytosed via a clathrin and dynamin-dependent route in activated T-cells [34]. According to Johanns et al. [35], up-regulation of *CTLA4* in regulatory T cells restrains effector T cell activation at early infection time points and allows the increase of bacterial burden during murine salmonellosis. Similarly, Inoue et al. [33] state that CTLA4-mediated Treg immunosuppression is critical in preventing the host from eliminating invasive pathogens. Given that, CTLA4 down-regulation, concurrent with clathrin up-regulation after the bacterial challenge, could indicate the repression of a mechanism of T cell inhibition in porcine MLN upon *Salmonella* Typhimurium infection. However, since clathrin could be involved in the establishment of both host immunity mechanisms and virulence strategies evolved by the pathogen, a deeper investigation of processes mediated by this molecule during infection is necessary and could provide relevant knowledge on the pathogenesis of porcine salmonellosis.

Current results also pointed to the generation of adaptive immunity mechanisms in infected tissue at a short time after infection. High MHCI levels observed by Western blot at 1 dpi reinforce our previous evidence that *Salmonella* antigens are cross-presented in swine MLN at initial stages of infection [10]. Cross-presentation is a mechanism that enables antigen presenting cells to prime CD8+ T cells via their own MHCI molecules [36]. Interestingly, it has been reported that differently from MHCII, *Salmonella* is not able to reduce MHCI surface expression of infected cells and consequently avoid early host cytotoxic response [28-30]. Therefore, cross-presentation might lead to an early *Salmonella* Typhimurium clearance by cytotoxic T cells during porcine infections, in agreement with the stimulation of *Salmonella*-specific CD8 T cells readily observed after mice and human infections [37]. Additionally, *CD180*, an inducer of B cell proliferation, activation and differentiation [38], was uncovered to be up-regulated all along infection. Taken together, our results indicate that both cellular and humoral immunity mechanisms are effectively engendered in porcine MLN at a short time after infection with *Salmonella* Typhimurium. Thus, the dynamics of this protective response could be decisive in the course of infection by this pathogen in pigs.

Evidence of pyroptosis induction and apoptosis dampening in infected MLN were disclosed in the current study, supporting our previous reports [10]. Thus, microarray data mining detected an enrichment of processes such as “Negative regulation of apoptosis” and “Antiapoptosis” after the bacterial challenge, in addition to up-regulation of genes encoding for an inhibitor of apoptosis proteins (IAP) like XIAP and PDCL3. Induction of apoptosis has been asserted as a strategy that facilitates *Salmonella* cell-to-cell spread during systemic infection [39]. Nevertheless, it has also been reported that AvrA, a *Salmonella* effector protein, prevents the apoptotic elimination of host cell niche as a pathogen evasion mechanism [40]. Intriguingly, we observed in vivo expression of *spvB* and *sseL*, both major *Salmonella* Typhimurium apoptosis inducers, and the apoptosis inhibitor *avrA*, indicating that *Salmonella* appeared to execute virulence mechanisms to modulate apoptosis in porcine MLN in its favor.

As in apoptosis, pyroptotic cells show DNA fragmentation, nuclear condensation and positive TUNEL staining [41,42]. However, pyroptosis inherently results in inflammation due to caspase-1-mediated maturation of pro-IL-1β and pro-IL-18 and release of the cytoplasmic content, whereas the apoptotic cell is considered to be immunologically silent [43]. In the current study, increase of TUNEL positive labeling was observed in the tissue at 1 dpi, as well as infiltration of inflammatory cells and IL1β up-regulation. Therefore, we propose that once apoptosis is dampened, the infected cell undergoes pyroptosis in swine MLN, producing pathogen discharge to the extracellular milieu and clearance of bacteria by innate mechanisms. In support of this, we previously reported an increase of phagocyte counts and mRNA levels of pro-inflammatory genes upon infection with *Salmonella* Typhimurium and a significant reduction of the pathogen burden at 6 dpi [21].

In line with this, an elegant study by Miao et al. [44] stated that *Salmonella* Typhimurium is able to damper pyroptosis for its own advantage by avoiding flagellin expression during infection of mice. Interestingly, we found expression of *Salmonella* Typhimurium flagella component (FliC) and regulators (FliA and FljA) in infected MLN. Additionally, flagella expression by infecting bacteria found in tissue was also corroborated by labeling using a specific polyclonal antibody. *Salmonella enterica* alternately expresses two different flagellar filament proteins, FljB and FliC, in a process known as flagellar phase variation. In spite of the high homology level found between these proteins, their middle surface exposed sequences of amino acids are divergent, resulting in distinct antigenicities [45]. It is noteworthy that our results demonstrate higher expression levels for *fliC* and its regulator *fliA* in vitro than in vivo. On the contrary, *fljA*, which is cotranscribed with *fljB,* was more expressed in *Salmonella* Typhimurium found in vivo. Moreover, this gene was notably less expressed than *fliA* and *fliC* in both early and late logarithmic phase cultures. Based on this, we deduced a skewing toward FliC flagellin expression by bacteria in vitro. Besides, we drew the inference that a more heterogeneous flagellin expression is found in *Salmonella* Typhimurium replicating in vivo and that induction of flagellar phase variation could be a strategy adopted by this pathogen to hinder pig immune response. Expression of prg*J* was also uncovered in swine MLN. Curiously, repression of this effector has been reported as a mechanism of pyroptosis inhibition in vivo [46]. Thus, it could be inferred that expression of flagellin and *prgJ* by *Salmonella* Typhimurium found in tissue might enable pigs to use pyroptosis to clear bacteria in gut associated lymph-nodes, protecting itself from pathogen dissemination. Nevertheless, an issue that should be addressed by our assumption is why pathogen burden in tissue peaks after pyroptosis triggering. Miao and Rajan [46] stated that in a single cell, pyroptosis only takes place at late times of infection, following bacteria replication. So, we inferred that increase of pathogen load at 2 dpi may be due to the release of replicated *Salmonella* from cells dead by pyroptosis.

Notably, the presence of TUNEL positive cells in MLN was significantly reduced at 2 and 6 dpi, suggesting a decrease of cell death by apoptosis or pyroptosis. As with any physiological process, excessive pyroptosis is detrimental to the host [41]. So, modulation of this pathway by the host aiming to restore tissue integrity should be expected. Actually, we observed up-regulation of *MAP3K7* and *TRAF7*, both involved in NF-kB and survival pathway activation, at 2 and 6 dpi. However, evidence indicates that inhibition of caspase-dependent apoptosis primes cells towards programmed necrosis [47]. Since the mechanisms that dictate the cellular decision to survive by activating NF-kB or to die through apoptosis or necroptosis are still unclear [48] further research is necessary to clarify these results.

In conclusion, the results provided led us to infer that although the *Salmonella* Typhimurium strain employed in this study was able to express some of its major virulence effectors in porcine MLN, a combination of early host triggered innate and adaptive immunity mechanisms might overcome virulence strategies employed by pathogens. Besides preventing apoptosis, swine appear to take advantage of flagellin and prgJ expression by pathogens to induce pyroptosis in MLN. In this context, pyroptosis might consist in a host protective mechanism that prevents pathogen spread beyond gut-associated lymph-nodes. Furthermore, cross-presentation of *Salmonella* antigens in MLN might result in a rapid clearance of pathogens by cytotoxic T cells. Functional relevance was also shown by clathrin-mediated endocytosis that could contribute to mechanisms of pathogen virulence and/or host defence in MLN of *Salmonella* infected swine. Further analysis of examined mechanisms may support the discovery of novel strategies of host defense against *Salmonella* at the intestinal level.

## Competing interests

The authors declare that they have no competing interests.

## Authors’ contributions

RPM was responsible for the whole study, including lab work, data analysis and interpretation, as well as the writing of the manuscript. CA participated in the confocal analysis. JEG collaborated with SCOTS analysis. AC performed the experimental infection and collected the tissue samples. RB and MGC performed microarray data processing. JJG conceived and designed the project and participated in the interpretation and discussion of the results, as well as in the writing of the manuscript. All authors read and approved the final manuscript.

## Supplementary Material

Additional file 1 **Primer pairs employed in host differential expression analysis by real-time quantitative PCR.** Table showing sequences and accession numbers of primers used for differential expression analysis in porcine mesenteric lymph-nodes. Click here for file

Additional file 2 **Primer pairs employed in the analysis of *Salmonella *Typhimurium virulence genes expressed in vivo.** Table showing sequences and accession number of primers used for quantification of *Salmonella* Typhimurium transcripts expressed in porcine mesenteric lymph-nodes. Click here for file

Additional file 3 **Differentially expressed transcripts.** Table showing chip ID, gene name, fold change and Bayes Factor value of transcripts differentially expressed in swine mesenteric lymph-nodes. Click here for file

Additional file 4 **Ingenuity Pathway Analysis annotations.** Excel spreadsheet describing networks, biofunctions and canonical pathways found to be enriched in swine mesenteric lymph-nodes after *Salmonella* Typhimurium infection. Click here for file

Additional file 5 **DAVID Bioinformatic Database analysis of genes involved in Cell Death.** Excel spreadsheet describing Gene Ontology terms associated to gene data set involved in Cell Death. Click here for file

Additional file 6 **Detection of *Salmonella *Typhimurium in mesenteric lymph-nodes of infected swine.** Video showing confocal analysis (Z-stack tool) of *Salmonella* Typhimurium labeled in mesenteric lymph nodes of infected swine. Click here for file
