# Supplementary material for: Pyroptosis and adaptive immunity mechanisms are promptly engendered in mesenteric lymph-nodes during pig infections with Salmonella enterica serovar Typhimurium
Source: Vet Res. 2013 Dec 5;44(1):120. doi: 10.1186/1297-9716-44-120 (PMC4028780; doi:10.1186/1297-9716-44-120)
Supplement: Additional file 1 — Primer pairs employed in host differential expression analysis by real-time quantitative PCR. Table showing sequences and accession numbers of primers used for differential expression analysis in porcine mesenteric lymph-nodes. [file 1297-9716-44-120-S1.doc]

| **Gene Name** | **Foward Primer (5’ → 3’)** | **Reverse Primer (5’ → 3’)** | **Accesion number** |
| --- | --- | --- | --- |
| **βActin** | CAGGTCATCACCATCGGCAACG | GACAGCACCGTGTTGGCGTAGAGGT | U07786 |
| **CASP1** | CTCTCCACAGGTTCACAATC | GAAGACGCAGGCTTAACTGG | NM_214162 |
| **CASP3** | CCTGGCGAAATTCAAAGGAC | CTTGTGAACATACTGTTTCAG | NM_214131.1 |
| **CD180** | GGGCAGCTTGGAGATTCTGA | GGAATGCTTGCTGGTCTATGG | NM_214357.1 |
| **CD1A** | TGAAGCACAGCAGCCTAGGA | GGAGCTGTGCTGTTCCCAGTA | AB189909.1 |
| **CTLA-4** | CCACCGAAAACAAAGTGAACCT | TTGCAGATGTAGAGCCCAGTGT | NM_214149.1 |
| **DAB2** | TTCAATGGTCACGGGTGCTA | CCGTTGGGCTTGTGGTATAGA | XM_003133906.3 |
| **EIF4H** | CAGACACCGCTTCCTACCAAA | GGTGGGAGGCACAGTATCTCA | NM_001243447.1 |
| **ENPP6** | TGTCAGCGATGCTCTTGACTTC | CAATGCGCTCGTGGTATATGG | AY609869.1 |
| **F13A1** | TCTCAGCCCATGACAACAATG | TCACGTTCCCATCTTCTTCCA | XM_001927630.4 |
| **HSPA1B** | GCCGAGAAGGACGAGTTTGA | TGGTACAGTCCGCTGATGATG | NM_213766.1 |
| **HSPH1** | TGGTGTGGAACCATGATTCAGA | CAGCGTGGTTCCTGCTAAACA | NM_001097504.1 |
| **IL16** | CGAAGACCCAGGTGCAAATAGT | CCGAAAGGTTGAGCGAGAAG | NM_213751.1 |
| **LPCAT2** | TTGGCCTGGCTGTCTTGTG | GCTTAAATGCCACCTGGATGA | XM_003126977.1 |
| **PSMC2** | CCAGCTGGACGGGTTTGAT | TCAGGTCTGTTTGTGGCCATT | NM_001245008.1 |
| **SLA-B** | CAGCTCTCGGCTACTACAACCA | TCGCAGCCGTACATGATCTG | AB205147.1 |
| **SLA-DRB5** | GTGACGGTGTATCCTGCAAAGA | ACAGAGCAGACCAGGAGGTTGT | NC_010449.4 |
| **TRAC** | TCCGCATCATCCTCCTGAA | ACCACAGCCGCAGTGTCAT | AB087982.1 |
