# Supplementary material for: Pyroptosis and adaptive immunity mechanisms are promptly engendered in mesenteric lymph-nodes during pig infections with Salmonella enterica serovar Typhimurium
Source: Vet Res. 2013 Dec 5;44(1):120. doi: 10.1186/1297-9716-44-120 (PMC4028780; doi:10.1186/1297-9716-44-120)
Supplement: Additional file 2 — Primer pairs employed in the analysis of Salmonella Typhimurium virulence genes expressed in vivo. Table showing sequences and accession number of primers used for quantification of Salmonella Typhimurium transcripts expressed in porcine mesenteric lymph-nodes. [file 1297-9716-44-120-S2.doc]

| **Gene Name** | **Foward Primer (5’ → 3’)** | **Reverse Primer (5’ → 3’)** | **Accesion number** |
| --- | --- | --- | --- |
| **avrA** | ACTGGCGTTGAGGACCAAAG | ACCACACAGACGTTCACAAATA | gi|16763390:c3010806-3009898 |
| **fliA** | GAGCTCTTCCTGGTAATACAGCGT | GCATCGAACTGGTGACTGAAGAAC | gi:380463450:c 2001677-2002396 |
| **fliC** | CCAGGTCAGAACGTAACGTGTCAA | GCAAGTAAAGCCGAAGGTCACAAC | gi:380463454:c 2004587-2006074 |
| **fljA** | TTCCTACGGGCGAGAAGCT | GAACTCGACGAGCAAGCATAGA | gi|378448274:c2933397-2932858 |
| **gyrA** | CTTTATCGTCTCCCGCCAGCTT | CGTTGACCTGACTTCTGGTTCT | gi|16445344:c2373711-2376423 |
| **prgJ** | CGGACATTGTCTCGCTGGAT | CAATCGCCGAACCAGAAAA | gi|378448274:c3036293-3035988 |
| **sifA** | CCAGCACCAGCCAAAGTATGT | GGCGTGAAAAACCTGATCAGA | gi|378448274:c1269017-1268007 |
| **sopB** | GCTGGGTTGGCGAATGGCTG | AATCCATCATCCCTGTACGA | gi|16763390:c1179916-1178231 |
| **spvB** | GCCGGCGAGCATATCTATTACT | TCCCATTGAGGTCCACATTGT | gi|378448040:c29958-28183 |
| **sseL** | CCCAACGGCTGTGGTCTATT | GATCGTTTTGTCCGGCATTC | gi|378448274:c2446348-2447301 |
